# The OptAIDS project: towards global halting of HIV/AIDS

**DOI:** 10.1186/1471-2458-9-S1-S1

**Published:** 2009-11-18

**Authors:** Robert J Smith?, Richard Gordon

**Affiliations:** 1 Department of Mathematics and Faculty of Medicine, The University of Ottawa, 585 King Edward Drive, Ottawa ON K1N 6N5, Canada; 2 Department of Radiology, University of Manitoba, Room GA216, HSC, 820 Sherbrook Street, Winnipeg MB R3A 1R9 Canada

## Introduction

We face a unique, transitory opportunity in the history of the HIV/AIDS epidemic, because we have collectively pooled money faster than the epidemic has grown [1]. Can we then seize the moment and halt this epidemic now? Most scenarios for the future of HIV/AIDS project modest reductions spread out over decades [2]. The very timescale of such projections, beyond the persistence time of all models, makes them unreliable [3]. Can we do better, quicker?

The OptAIDS project was conceived as a means to address this issue. Its implementation thus far has been twofold: a workshop held in July 2008 and this supplement on the eradication of AIDS. The aims of the project are to address two questions:

1. Can we optimally spend our way out of the HIV/AIDS epidemic?

2. Can we work together to build a World Halting AIDS Model (WHAM) that would permit us to estimate the quickest way to halt HIV/AIDS, monitor our success, and adjust our strategy as we go?

The OptAIDS project grew out of a frustration with existing attempts to tackle the disease. AIDS exceptionalism means that HIV/AIDS is handled differently from other public-health epidemics, which has likely been detrimental [4,5]. Consequently, much of the funding of HIV/AIDS efforts has been for qualitative observations of the expanding epidemic rather than quantitatively effective intervention.

Although fund accumulation has recently outpaced the epidemic, we argue that plans to spend donor money are too long range in the face of a growing epidemic [6]. Long-range scenarios have no reality to them, so that only short-term solutions - those that fall within the persistence time of their models - have any possibility of being realistic [3]. Furthermore, disease is a global problem that is only tackled locally [7]; epidemics cross borders, whereas we fund mostly local or national "solutions".

The OptAIDS project was an outgrowth of the Stop Afghan AIDS project [8]. This project was led by mathematical modellers planning to continuously adapt their models to new data and predicting what data should be collected. The Stop Afghan AIDS project showed how it should be possible to intervene quantitatively in an epidemic. The usefulness of modelling in complex systems is not new. Mathematical models of the economy tell us whether a decrease in income tax will result in an increase in investment or an increase in imported consumer goods. Mathematical models of the atmosphere tell us what the effects of carbon dioxide emissions or of nuclear wars may be. Mathematical modelling is used routinely in such things as aircraft design and the design of traffic systems [9].

So too, epidemics are quantitative creatures with predictable thresholds. Models that can be adapted to new results and to changes in control policy have been identified as an integral part of disease-control programs [10]. Modelling-led interventions were instrumental in halting the 2001 Foot and Mouth outbreak in the UK [11]. A mathematical model of the dynamics of measles in New Zealand developed in 1996 successfully predicted an epidemic in 1997 and was instrumental in the decision to carry out an intensive immunisation campaign in that year. While the epidemic began some months earlier than anticipated, it was rapidly brought under control, and its impact on the population was much reduced [12].

The West African Onchocerciasis (river blindness) Control Program successfully used modeling to supplement intervention programs [13]. By using clearly delineated endpoints, these models helped convince donors and the scientific community that the aims of the program were achievable [14]. As a result, mathematical models have retained a role in subsequent policy discussions [15]. Insights from mathematical models during the SARS epidemic helped determine how serious the epidemic might become, as well as the impact of proposed control measures. These models provided important guidance to public-health authorities at a critical time when little other information was available. Insights from the models showed that, if unchecked, the virus could cause a significant epidemic, but that basic epidemiological control measures - patient isolation, contact tracing, etc - could have a substantial impact on the extent of the epidemic. Subsequently, these control measures played a major role in limiting the spread of the 2003 epidemic [16].

Weather prediction models provide a workable analogy. Such models consist of continually updatable inputs, that must adapt to an enormous array of incoming data [17]. Short-term predictions, especially those associated with discrete, extreme weather events such as floods and hurricanes, have proven useful in supporting emergency management strategies, unlike events such as earthquakes or acid rain, which have longer lead times [18]. Complex mediating models which themselves have explanatory power and which embody techniques of modeling can be refined and passed down to successor models [9]. The virtue of mathematics in such a context is that it forces clarity and precision upon the conjecture, thus enabling meaningful comparison between the consequences of basic assumptions and the empirical facts [19].

Existing scenarios for HIV control have typically been spread out over two or more decades [20], which means that the reliability of their predictions is low. The basic concept of OptAIDS is to spend more money up front, effectively, based on the best models and their parameters we can formulate, with the goal being a rapid halt to the epidemic with the fewest additional cases. This means that models can be shorter term and therefore more reliable, because we stay within the models' persistence time. OptAIDS emphasises continuous monitoring to check the accuracy and adjust the parameters of the global model. Mathematically, this is an optimal halting problem.

## The workshop: Real Life and Second Life

The OptAIDS workshop was the first of its kind: a scientific meeting held simultaneously in both a real world location and also Second Life^® ^http://secondlife.com, a virtual landscape that allows real-time communication. The broad topic was the eradication of AIDS using optimal spending models, but this encompasses an enormous number of issues surrounding the AIDS epidemic. Topics covered included the impact of circumcision, the effect of traditional medicine, prevention strategies for countries with nascent epidemics and the difficulties of developing an HIV vaccine.

MITACS http://www.mitacs.ca gave us a Can$10,000 workshop grant for this meeting, which was held on July 29, 2008. Given that this amount would only cover a few airfares, we decided to allow people to participate via Second Life^®^. Second Life^® ^allows the creation of avatars [21], so that users can participate in the world. Within the virtual world, you can talk to other people's avatars, upload PowerPoint slides and manipulate objects within the environment.

**Figure 1:**
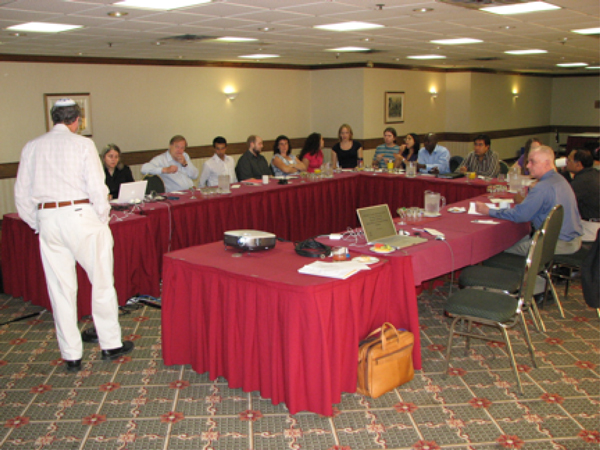
**OptAIDS participants in Toronto, July 29, 2008**.

**Figure 2:**
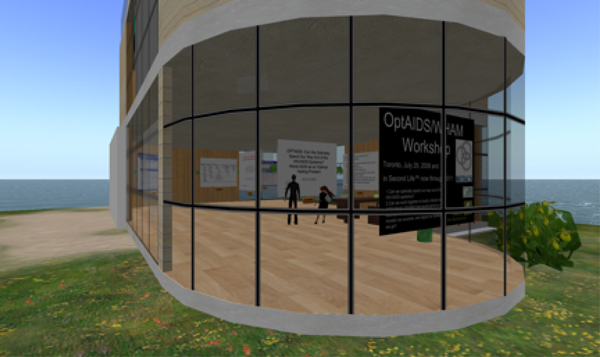
**Posters from the OptAIDS Workshop may be viewed at http://slurl.com/secondlife/Silver Bog/21/27/22**.

**Figure 3:**
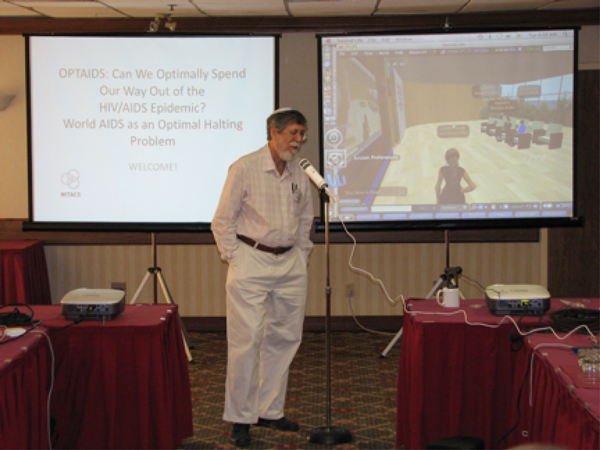
**Richard Gordon introducing the OptAIDS Workshop in Toronto**. The screen on the right shows the scene in Second Life, where remote participants viewed the same slides on a virtual screen.

Twenty-two people convened over the day in Toronto (Figure 1), including ten presenters. The traffic count during the day indicated 500 avatar arrivals in Second Life^®^, four of whom were presenters. The workshop was advertised to pertinent groups in Second Life^® ^and open to the avatar public. The Second Life^® ^building includes a location where the original slide presentations can be viewed (Figure 2). Two screens were used in Toronto, showing the slides and the audience (represented by avatars), while Second Life^® ^participants could lecture by having their avatar stand near a virtual screen in Second Life^®^. Everyone could hear everyone else. A second virtual screen showed a live camera view of the audience in Toronto (Figure 3). The all-day meeting had only a few short interruptions for technical reasons. A summary and follow-up discussion was presented at the annual meeting of the Society for Mathematical Biology shortly afterwards.

The four speakers presenting via Second Life^® ^were located in Poland, Seattle, Denmark and Los Angeles. The technology allowed for interactive discussion, so speakers in Toronto faced questions from Second Life^® ^participants all around the world, while Second Life^® ^speakers had their Powerpoint presentations shown on a screen in Toronto (operated by their avatars in Second Life^® ^and simultaneously by the organisers in Toronto), and faced questions from Toronto and other Second Life^® ^participants. The size of the turnout in Second Life^® ^demonstrated the effectiveness of virtual conferencing; many more people were able to attend the conference than would have been feasible otherwise.

The event was covered by the *National Post*, which reported on the innovative use of Second Life^® ^in an academic setting. All the presentations remain in Second Life^®  ^http://slurl.com/secondlife/Silver Bog/21/27/22 as posters that can be clicked on by anyone interested. Speakers can be asked to show up personally as avatars to go over the slides.

## *BMC Public Health*: towards global halting of HIV/AIDS

The aim of this supplement is to discuss AIDS as a global phenomenon and address issues surrounding its eradication. Due to the scale of the epidemic, a great number of sub-issues arise. In thinking of AIDS as a global pandemic, we need to tackle the disease from as many directions as possible. Some of the articles involve mathematical models, others involve a thorough examination of the state of resources, or an understanding of the effect of the disease on society.

This supplement comprises fifteen articles (including this introduction), divided into six themes:

1. History

2. Resources

3. Demographics

4. In-host models

5. Computation

6. Spending our way out of the epidemic

Theme 1 comprises an introduction and overview of mathematical modeling [22], as well as a history of AIDS in Africa and its effects on human development [23]. Theme 2 is concerned with the various resources that comprise our intervention arsenal: the allocation of resources [24], cost-effectiveness of prevention [25], antiretroviral pricing [26], the effects of migration upon availability of health professionals [27], and the relationship between mathematical models and resource allocation [28].

Theme 3 looks at the effects of demographic changes in China on HIV [29] and the spread of HIV among men who have sex with men [30]. Theme 4 examines in-host modeling - a crucial element in tackling the disease, often overlooked by epidemiologists - by proposing new methods for evaluating the efficacy of antiretroviral treatment [31] and examining antioxidant supplementation as HIV therapy, with a focus on injecting drug users [32].

Theme 5 looks at using virtual epidemics to understand real ones [33] and develops an epidemic simulator of an agent-based, data-driven disease model [34]. Finally, Theme 6 examines the question at the core of the OptAIDS project: spending our way out of the AIDS epidemic [6].

The collection of articles in this supplement run the gamut of topics related to HIV/AIDS. They examine the disease from a global perspective, in an attempt to untangle many of the problems associated with the epidemic. However, we view this as a starting point: the next step is for policymakers and the donor community to embrace the idea of global eradication. Only by working together can we combat this disease.

## Conclusion

AIDS is the fourth worst infectious disease of all time, resulting in more deaths per day over the past 25 years than occurred on 9/11/2001 [35]. Over 33 million adults are now infected with HIV, many in the developing world, where resources are scarce and infrastructure is struggling under the weight of this burgeoning epidemic. The HIV/AIDS epidemic is often spoken of in terms of "reducing the spread" [36], or achieving "sustainable financing" [37]

However, this special issue demonstrates that, despite the immensity of the epidemic, eradication is not only possible, it is feasible. The time has come to stop thinking locally and to start acting globally.

## Competing interests

The authors declare that they have no competing interests.

## Authors' contributions

RJS wrote the introduction, overview of the supplement and the conclusion. RG set the theme in the first draft and wrote the start of the introduction and the section on the workshop. Both authors proofread and approved the final manuscript.
